# A Multiomic Study of Platelet-Derived Extracellular Vesicles and Impact of Platelet Concentrate Sources

**DOI:** 10.1049/nbt2/8358424

**Published:** 2025-08-19

**Authors:** Andreu Miquel Amengual-Tugores, Carmen Ráez-Meseguer, Maria Antònia Forteza-Genestra, Javier Calvo, Antoni Gayà, Marta Monjo, Joana Maria Ramis

**Affiliations:** ^1^Department of Fundamental Biology and Health Sciences, University of the Balearic Islands, Palma, Balearic Islands, Spain; ^2^Group of Cell Therapy and Tissue Engineering (TERCIT), Research Institute on Health Sciences (IUNICS), University of the Balearic Islands, Palma, Balearic Islands, Spain; ^3^Health Research Institute of the Balearic Islands (IdISBa), Palma, Balearic Islands, Spain; ^4^Fundació Banc de Sang i Teixits de les Illes Balears (FBSTIB), Palma 07004, Balearic Islands, Spain

**Keywords:** molecular cargo, multiomics analysis, platelet concentrates, platelet-derived extracellular vesicles, wound healing

## Abstract

Platelet-derived extracellular vesicles (pEVs) are a potent fraction of platelet concentrates, enhancing their therapeutic potential in regenerative medicine. This study evaluates pEV from three platelet sources: platelet lysate (PL), fresh platelets (fPs), and aged platelets (aPs), to determine how activation and storage conditions affect pEV characteristics, functionality, and molecular content. pEV are isolated using size exclusion chromatography (SEC) and characterized by transmission electron microscopy (TEM), western blot, and nanoparticle tracking analysis (NTA). Functional assays include wound healing, metabolic activity, and cytotoxicity. Protein and miRNA profiles are obtained through LC-MS/MS and miRNA arrays, followed by bioinformatic analysis. Findings show that PL-derived pEV exhibits the highest yield and purity, containing markers CD63 and CD9. Enhanced fibroblast migration in wound healing assays suggest a critical role for PL-pEV in hemostasis, proliferation, and remodeling phases. Multiomics analysis identifies upregulated miRNAs, particularly miR-210-3p and the miR-320 family, associated with wound healing. Differential protein analysis reveals an enrichment in immune response and wound healing pathways within PL-pEV. These results demonstrate the impact of platelet preparation methods on pEV molecular cargo and efficacy, with hsa-miR-320a, hsa-miR-320b, and hsa-miR-210-3p identified as key mediators supporting the clinical potential of PL-pEV in regenerative medicine.

## 1. Introduction

Extracellular vesicles (EVs) first description dates back to 1967, when Peter Wolf reported the release by platelets of minute lipid-rich particulate that termed as “platelet dust.” Nevertheless, and although platelet concentrates are broadly used in regenerative medicine [1], their therapeutic effect has only recently been attributed to the EV that platelets release (platelet-derived extracellular vesicle [pEV]). Specifically, the use of pEV has been proposed as an emerging therapeutic asset for its beneficial activity demonstrated in wound healing, muscle and skeletal tissue regeneration, angiogenesis, and neural regeneration [2–8].

Using platelets as EV source in therapeutics presents several advantages compared to other cell sources. For instance, pEV can be directly obtained from whole-blood donations without the need of ex vivo expansion, easing the industrial and regulatory challenges for its clinical translation [9]. However, platelet concentrates source and downstream processing to obtain pEV may determine their characteristics, activity, functionality, molecular cargo, and the capability of being used in the clinical field [10, 11]. For example, platelets can be activated by the addition of CaCl_2_, by the use of bovine thrombin or through platelet aging [12], which is resting the platelet concentrates before pEV isolation [13, 14]. Also, it has been described that by freeze-thawing the platelet concentrates a so-called platelet lysate (PL) is obtained, being equally effective for the release of active biomolecules. Another advantage compared to other EV sources is that donor-to-donor variability can be decreased by pooling platelet donors [15, 16]. Despite the promising results in the biopharmaceutical field, there is no consensus on the optimal source of pEV to be used as an active compound in regenerative medicine.

To answer this question, we evaluated pEV obtained from three different platelet concentrates—PL, fresh platelet (fP) concentrates, and aged-platelet (aP) concentrates—by means of their characteristics, functionality, and molecular cargo through multiomics analysis.

## 2. Materials and Methods

### 2.1. Platelet Concentrates Procurement

Platelet concentrates were provided by the IdISBa Biobank, with the approval of the Ethics Committee (IB1955/12 BIO ref. 02/2021) after ethical approval of the project by the CEI-IB (IB 4453/21 PI). In brief, six buffy coats were pooled, washed with NaCl 0.9% (Braun Melsugen AG, 34209 Melsugen, Germany), and centrifuged at 651 × *g* for 10 min obtaining the platelet concentrates. Three different types of samples were obtained afterwards: (1) PLs, (2) fP concentrates, and (3) aP concentrates.

PL was generated by applying three freeze/thaw cycles (−80/37°C) to the platelet concentrate and a freeze/thaw cycle (−80/55°C) for pasteurization; followed by a centrifugation at 5050 × *g* for 20 min and filtered through 40 μm porous membrane filters (Sartorius). Then, PL were centrifuged, first at 1500 × *g* for 15 min at 4°C and second at 10,000 × *g* for 30 min at 4°C, keeping the supernatant after each centrifugation. Subsequently, the supernatant was filtered successively through 0.8 and 0.2 µm porous membrane filters (Sartorius, Goettingen, Alemania).

To obtain fP, one tenth of the volume of coagulation activator (2.3% CaCl_2_ prepared in AB serum) was added to the platelet concentrate. Subsequently, it was incubated for 20 min at 37°C and for 15 min at 4°C. After that, it was centrifuged at 5050 × g for 20 min, thus, obtaining the fP supernatant. Improvement: The same procedure was employed for obtaining aP, with the added step of maintaining the platelet concentrates under agitation in an agitator (Helmer) for 7 days at 25°C.

### 2.2. pEV Isolation

pEVs from the different platelet concentrates were isolated by size exclusion chromatography (SEC), loading 10 mL of each platelet concentrate into the chromatography loop. A commercial column (HiPrep 26/60 SephacrylS 400HR, AKTA, USA) connected to an ÄKTA go system (Cytiva, USA) coupled with a fraction collector F9-R (Cytiva) was set to allow a flow rate of 1.3 mL/min using 0.9% NaCl (Braun). Thirty fractions of 5 mL each were collected and characterized. Fractions 8, 9, and 10 were pooled and used as pEV preparations. This process was carried out in the clean room of the Fundació Banc de Sang i Teixits de les Illes Balears. pEVs were characterized following MISEV2023 recommendations [1].

### 2.3. Nanoparticle Tracking Analysis (NTA)

The number of particles and their size distribution was analyzed using NTA with Nanosight NS300 (Malvern Instruments, Malvern, UK). Data were analyzed with NTA 3.2 Dev Build 3.2.16 software, taking five videos of 25 s per sample. A green laser was applied with a camera level of 16 and the videos were taken at a temperature of 25°C. The analysis was carried out with a detection threshold of 4. The quantification by number of particles per ml allowed to calculate the purity in terms of number of particles per µg of protein and the yield in number of obtained particles per mL of platelet concentrate.

### 2.4. Transmission Electron Microscopy (TEM)

PL-EV, fP-EV, and aP-EV were prepared following the protocol by Corona et al. [17], using glutaraldehyde (Sigma Aldrich, Germany), formaldehyde (Sigma Aldrich, Germany), and grids (Ted Pella, Redding, California, USA). Uranyless (Em-grade, France) Aqueus Solution was used in the staining step. Samples were observed by using the TEM-H600 (Hitachi, Tokyo, Japan) at 50 kV.

### 2.5. Protein Quantification

Total protein content was determined using the Pierce BCA Protein Assay Kit (ThermoScientific, Waltham, MA, USA) following manufacturer's protocol.

### 2.6. Western Blot

The pEV samples and their respective sources were prepared with nonreducing loading buffer (without β-mercaptoethanol) to detect tetraspanins presence and the same amount of protein (2.5 µg) was loaded in a 12% SDS-PAGE gel. For HSC70, albumin, cytochrome C (CytC), and ApoA detection pEV samples were prepared with reducing loading buffer (with β-mercaptoethanol), loading 15, 5, 5, and 15 µg, of protein, respectively, in a 10% SDS-page gel and 12% for CytC. Proteins were transferred onto nitrocellulose membrane (GE Healthcare, Pittsburgh, PA, USA) by humid transference. In order to confirm the correct transferring of the proteins.

For CD63 and CD9, membranes were blocked with 10% dry skimmed milk (Central Lechera Asturiana, Asturias, Spain) in TBS containing 10% Tween-20 (Scharlab, Barcelona, Spain) and incubated overnight at 4°C with the following primary antibodies: antihuman CD9 monoclonal antibody (clone Ts9 diluted 1 : 2,000, Thermo Fisher) and antihuman CD63 monoclonal antibody (clone TS63, diluted 1 : 2000, Abcam, Cambridge, UK). Then, membranes were incubated for 1 h with HRP-coupled secondary antibody (Thermo Fisher) diluted 1:2000. For membrane exposure, membranes were incubated with Clarity Western ECL Substrate (Bio-Rad, Hercules, CA, USA). GS-800 Calibrated Densitometer (Bio-Rad), was used for membrane exposure and image processing.

For HSC70, CytC, ApoA, and albumin detection, membranes were blocked with TBS Odyssey Intercept Blocking Buffer (LI-COR, Lincoln, USA) and incubated overnight with antihuman HSC70 mouse monoclonal antibody (clone B-6 diluted 1:2500, Santa Cruz), antihuman CytC mouse monoclonal antibody (clone 37BA11 diluted 1:1000 Abcam), antihuman ApoA mouse monoclonal antibody (clone 513 diluted 1:4000, Invitrogen), and antihuman albumin mouse monoconal antibody (cloneAL-01 diluted 1:2000, Invitrogen). Then, all the membranes were incubated for 1 h with IRDye 800CW Donkey anti-Mouse (diluted 1:8000, LI-COR) secondary antibody. Odyssey Infrared Imaging System scanner was used for membrane exposure and image processing.

### 2.7. Cell Culture

Immortalized human gingival fibroblasts-hTERT-hNOF (ihGF, Applied Biological Materials Inc), grown at 37°C and 5% CO_2_, were used in cell culture experiments. Cells were maintained in Dulbecco's modified eagle's medium (DMEM) without magnesium and calcium (Gibco, Grand Island, NY, US), Ham's F12 (Biowest, Nuaille, France), fetal bovine serum (FBS, Biowest), 100 µg/mL penicillin, and 100 µg/mL streptomycin (Biowest).

### 2.8. Wound Healing Assay

In vitro wound closure assays were carried out as previously described in another study from our group [18]. In brief, cells were seeded in triplicates at a density of 30,000 cells per well in a 48-well plate in three independent experiments. After 3 days, when cells had reached confluence, cell medium was replaced by medium containing 1% EV's-depleted FBS (obtained by previous ultracentrifugation at 120,000 × *g* for 18 h at 4°C). After 24 h, wound was performed using a sterile pipette tip, images were taken using a bright-field inverted microscope (Nikon Eclipse TS100) and cells were treated with 1 × 10^10^ particles of PL-EV, fP-EV, or aP-EV per well. After 24 h, the imaging was repeated.

Images were analyzed with ImageJ software (NIH, Bethesda, Maryland, USA) using a wound healing assay plugin [19]. Images were filtered at 32 pixels in variance method and wound area were obtained. The percentage of wound closure was calculated using the following equation:

  $$\begin{array}{c} \text{Wound closure }\left(\%\right)=\frac{\left(\text{Wound area at }0\;h-\text{wound area at }24\;h\right)}{\text{Wound area at }0\;h}\times 100. \end{array}$$

### 2.9. Metabolic Activity Assay

To measure metabolic activity 24 h after treatment with pEV, we used the Presto Blue reagent (LifeTechnologies/Thermo Fisher Scientific, Basel, Switzerland) following the manufacturer's instructions. Control cells (nonwounded and nontreated) were considered as 100% of metabolic activity.

### 2.10. Cytotoxicity

To determine the biocompatibility of pEV, lactate dehydrogenase (LDH) activity released to cell culture media was measured with the commercial Cytotoxicity Detection kit (Roche Diagnostics, Manheim, Germany) following the manufacturer's instructions. For cytotoxicity calculation, cells treated with 0.1% Triton X-100 were used as a high control (100% of cell death), and control cells (nonwounded and nontreated) as a low control (0% of cell death). The percentage of cytotoxicity was calculated using the following formula:

  $$\begin{array}{c} \text{Citotoxicity }\left(\%\right)=\frac{\left(\text{Sample absorbance}-\text{low control absorbance mean}\right)}{\left(\text{High control absorbance mean}-\text{low control absorbance mean}\right)}\times 100. \end{array}$$

### 2.11. RNA Isolation

RNA isolation from PL-EV and aP-EV samples was performed using miRNeasy Serum/Plasma Kit (Qiagen, Hilden, Germany), according to the manufacturer's protocol. Prior to extraction, UniSp2, UniSp4, and UniSp5 RNA Spike-in Template Mix (RNA Spike-In Kit, Qiagen) were added to each sample as an internal control. Process efficiency and RNA size distribution was assessed by RNA 6000 nano kit (Agilent Technologies, Santa Clara, CA, USA) and Agilent 2100 Bioanalyzer (Agilent Technologies).

### 2.12. Microarray Analysis

For miRNA profiling analysis, previously obtained samples were labeled with the FlashTag Biotin HSR RNA labeling kit (Affymetrix, Santa Clara, CA, USA) according to the manufacturer's instructions. Biotin-labeled RNA molecules were incorporated into the GeneChip miRNA 4.0 Array (Affymetrix) and hybridized for 42 h at 49°C in the Hybridization Oven 645 (ThermoFisher). Arrays were washed and stained at the Fluidics 450 station (Affymetrix) with the GeneChip Hybridization, Wash, and Stain Kit (ThermoFisher) and subsequently scanned with the GeneChip Scanner 3000 system (Affymetrix). miRNA expression profile was analyzed with the transcriptome analysis console software (ThermoFisher). These differentially expressed miRNAs were then validated in the entire series using quantitative real time polymerase chain reaction (RT-PCR).

A total of 1.12 µL of eluted RNA were reverse transcribed into cDNA using miRCURY LNA RT kit (Qiagen). Subsequent quantitative RT-PCR reactions were performed using the hsa-miR-320c miRCURY LNA miRNA PCR Assays (MIMAT0005793: 5′AAAAGCUGGGUUGAGAGGGU, Qiagen; Real-Time PCR Light Cycler 480 II, Roche). Relative gene expression was normalized to U6 snRNA (v2) miRCURY LNA miRNA PCR Assays (YP02119464).

### 2.13. Organic Solvent Precipitation (OSP)

The extraction of protein from samples was performed as follows: Absolute methanol was added to each sample in a 70:30 proportion and centrifuged at maximum speed for 15 min at 4°C. The precipitates were resuspended with lysis buffer (Tris 50 mM, NaCl 150 mM, EDTA 1 mM, Triton X-100 1%, protease, and phosphatase inhibitors, Complete-Mini and Phospho-Stop, respectively). Protein concentration was measured through NanoDrop. Pure isopropanol was added to 20 μg of protein from each sample, vortexed vigorously and stored at −20°C overnight. The following day, samples were centrifugated at maximum speed, for 10 min and 4°C. Supernatants were removed and precipitates were left to dry on ice in a fume extraction hood. The pellets were resuspended in NH_4_HCO_3_ 50 mM and sonicated. Reduction of disulfide bridges was carried out by adding DTT for 30 min at 98°C. Then alkylation processes were performed with IAA for 30 min at room temperature. Protein digestion consists in adding trypsin and let o/n at 37°C. TFA 2% was added to the digested proteins, followed by the incorporation of sample buffer (2% TFA in 20% MeCN). Samples were derived to SpinColumns (PierceC18 Spin Columns), following the manufacturer's instructions and concentrated using SpeedVaac (Eppendorf, Hamburg, Germany). Samples were resuspended in H_2_O/AF 0.1% before sample acquisition.

### 2.14. Mass Spectrometry (Proteome) Analysis

Peptides were separated on a Thermo Scientific EASY-nLC 1200 system. LC-MS/MS analysis was performed by injecting 1 µL onto a precolumn (ThermoScientific PePMap NeO C18, 5 μm, 300 µm × 5 mm, 1500 bar) coupled directly to a self-packed analytical column (ThermoScientific DNV PePMapTM Neo C18, 2 µm, 75 µm × 500 mm, 100 Å, 1500 bar), followed by a gradient elution into the mass spectromether (Thermo Scientific QExactive-Orbitrap Ms) equipped with a nanospray flex ion source (NSI). A full scan acquisition was performed with a resolution of 70,000 mode over a range of 375–1500 *m*/*z*. The spectrometer was operated in data dependent mode where the top 15 most abundant ions in each MS scan were subjected to MS/MS in the HCD cell with normalized collision energy of 28, whose resolution was 17,500 with dynamic exclusion of 40 s.

The temperature of ion transfer capillary was set to 275°C and the spray voltage was set to 1.9 kV in positive mode and S-lens RF level 50 AU, respectively. Data processing was performed using Thermo Proteome Discoverer 2.4.1.15 software. Spectra were searched using Sequest HT. Peptide spectral matches (PSMs) to the reverse database were used to calculate a global false discovery rate and were discarded. Data were further processed to remove PSMs with an FDR greater than 1.0%.

### 2.15. Bioinformatical Analysis

Bioinformatic study of microarray and PSM-derived data was performed using RStudio software. Of the total number of miRNAs and proteins detected, those with a higher differential expression level (*p* < 0.05 and FC > 1.5) were selected. From this set, a list of top 10 miRNA and 50 proteins with superior statistical significance was established for further bioinformatic analysis.

Gene Ontology (GO) enrichment and Kyoto Encyclopedia of Genes and Genomes (KEGG) analysis were performed according to the expression profile of miRNA and proteins, between aP and PL-EV. Protein-enriched functions were analyzed through GSEA and ORA data bases. All multiomics analyses were performed with a sample size of *n* = 3, corresponding to three independent batches of pEV derived from 50 donors each. Additionally, for each batch analyzed, two technical replicates were included in the multiomics analyses.

### 2.16. Statistical Analysis

For EV characterization and in vitro assays, statistical analysis began with a homoscedasticity test, followed by the Shapiro–Wilk normality test. These tests were conducted for each dataset prior to selecting the appropriate statistical test. Differences between experimental groups were evaluated using one-way ANOVA followed by Tukey's post hoc test for normally distributed and homoscedastic data and the Mann–Whitney *U* test for nonparametric data. These statistical procedures were applied only to EV characterization and in vitro assay datasets; multiomics data analysis was performed using specific bioinformatics pipelines described in the corresponding sections. All multiomics analyses were performed with a sample size of *n* = 3, corresponding to three independent batches of pEV derived from 50 donors each. Additionally, for each batch analyzed, two technical replicates were included in the multiomics analyses.

## 3. Results

**Figure 1:**
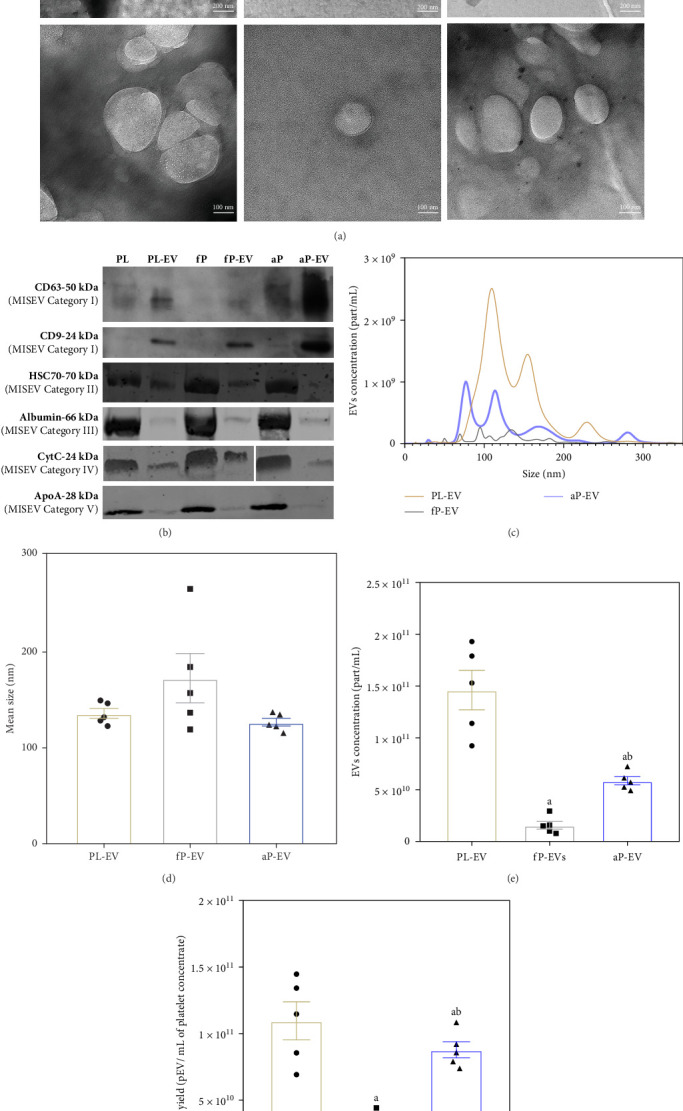
Characterization of PL-EV, aP-EV, and fP-EV by TEM, NTA, and western blot. (A) Representative TEM images for each group of EV enriched fractions from PL, fP, and aP at different magnification scales: 28kx and 94kx, with their respective scale bars. EV are shown as spheres. (B) Typical EV positive and negative markers, CD63 (50 kDa, MISEV Category 1), CD9 (24 kDa, MISEV Category 1), HSC70 (70 kDa, MISEV Category 2), albumin (66 kDa, MISEV Category 3), CytC (24 kDa when present as a dimer [20], MISEV Category 4), and ApoA (28 kDa, MISEV Category 5) identified by western blot from pEV samples isolated from PL, fP, and aP. The same amount of protein (2.5 µg) was loaded per well for canonical tetraspanins CD9 and CD63, 5 µg for albumin and ApoA markers, 10 µg for CytC, and 15 µg for HSC70. (C) Particle size distribution, (D) mean size, and (E) mean particle concentration determined by NTA analysis of pEV isolated from the different sources. (F) Mean pEV yield expressed as number of pEV particles per mL of platelet concentrate. Parametric data were evaluated by one-factor ANOVA with a Turkey post hoc. Significant differences were considered for *p*  < 0.05 and represented with “a” compared to PL-EV, or “b” compared to aP-EV. Sample size was *n* = 3, corresponding to three independent batches of pEV derived from 50 donors each.

The characterization results showed that fractions from 8 to 10 after SEC isolation from the three different platelet sources evaluated were enriched on pEV (Figure S1), so these fractions were pooled and further characterized by TEM (Figure 1A), western blot (Figure 1B), and NTA analysis (Figure 1). The evaluation of EV marker tetraspanins CD9 and CD63 and luminal EV marker HSC70, and negative markers albumin, CytC, and ApoA by western blot (Figure 1B and Figure S2) resulted in an enrichment of tetraspanins compared to their sources. These results were observed among all the groups.

While no differences in pEV size were observed among groups, higher pEV concentration (Figure 1E) was achieved for the PL-EV compared to the other two groups, in line with pEV yield (Figure 1F) showing, the fP-EV group, a markedly lower concentration hindering its clinical use. Thus, this group was excluded from further evaluation. Finally, particle concentration and protein content allow us to obtain a purity ratio. The mean purity obtained in the pEV-enriched fractions were 9 × 10^8^ ± 2 × 10^8^ particles/µg of protein for PL-EV, 1.37 × 10^8^ ± 1.00 × 10^8^ particles/µg of protein fP-EV, and 3.37 × 10^8^ ± 1.73 × 10^8^ particles/µg of protein for aP-EV. No statistical differences were observed when comparing the purity of these groups.

**Figure 2:**
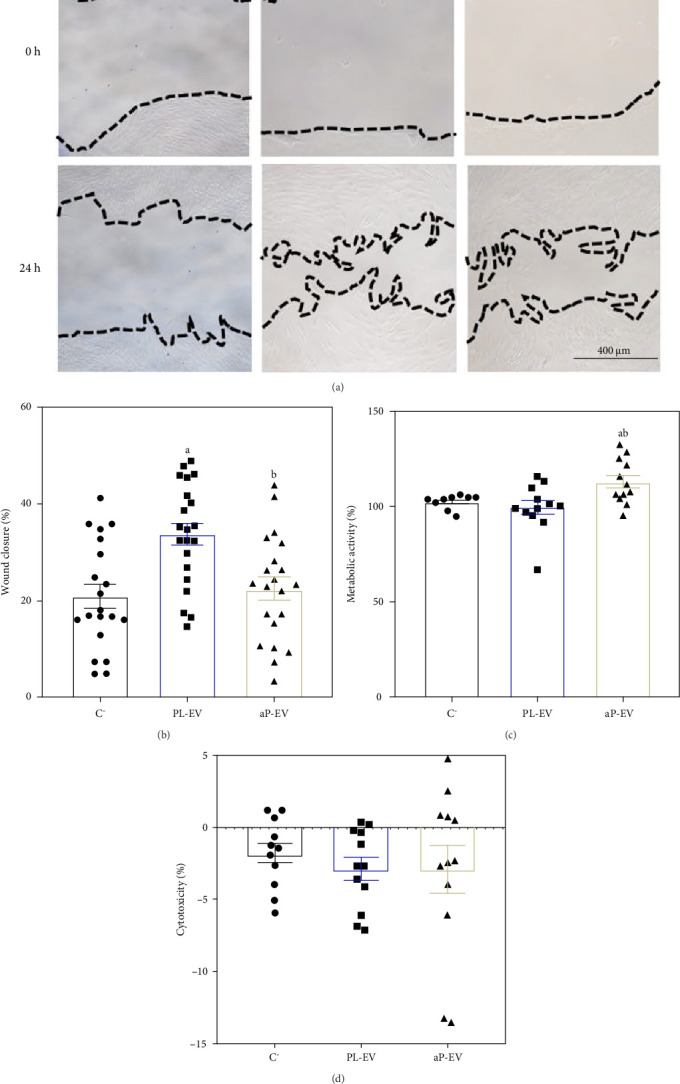
In vitro functional assay in hGF cells. (A) Representative images of wound healing assay at 0 and 24 h after treatment with PL-EV and aP-EV. C-, nontreated control. Wound area limit is indicated by dashed lines. (B) Wound closure area after 24 h of treatment. (C) Metabolic activity measured 24 h after treatment, C^−^ was set as 100%. (D) Cytotoxicity measured in culture media after 24 h of treatment; culture media from nonhealed cells was set as 0% of cytotoxicity and culture media from cells treated with 1% TritonX-100 was set at 100%. Values represent the mean ± SEM. Parametric data were evaluated by one factor ANOVA using DMS as a post hoc (B, D) and nonparametric data by *U* of Mann–Whitney test (C). Significant differences were considered for *p*  < 0.05 and represented with “a” compared to C^−^, “b” compared to PL-EV. fP-EV could not be tested in vitro due to the low particle amount obtained. Sample size was *n* = 3: each condition was assessed in three independent experiments, each comprising three technical replicates.

In the in vitro assays, we determined the wound closure area, metabolic activity, and cytotoxicity 24 h after treatment (Figure 2). Higher wound closure was achieved when cells were treated with PL-EV, compared to both: negative control (C^−^) and to cells treated with aP-EV. Furthermore, aP-EV increased the metabolic activity of treated cells compared to C^−^ and PL-EV. No cytotoxicity evaluated as LDH activity in cell culture media was observed for any experimental group, nor within the groups.

**Figure 3:**
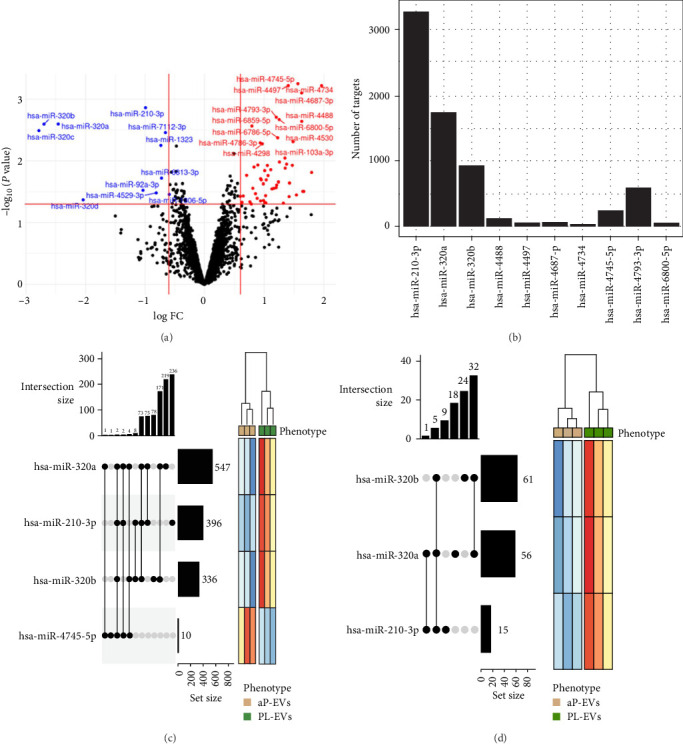
Differential expression profile analysis of miRNAs. (A) Volcano plot generated from microarray data. Each detected miRNA is plotted according to its statistical significance and fold-change value. Significant up- and downregulated miRNAs (*p*  < 0.05; FC > 1.5) are shown in red and blue dots, respectively, in aP-EV compared to PL-EV samples. miRNAs with no noticeable differences are shown as black dots. (B) Number of gene targets identified for each of the top 10 miRNA with superior statistical significance. (C) UpSet plot from Gene Ontology (GO) enrichment analysis and (D) KEGG pathway analysis. Set size indicates the number of identified GO or KEGG terms, respectively, associated with each of the miRNAs included in the top 10 list. Interaction size reflects the number of terms shared by these miRNAs (connected dots). The associated heat map is shown on the right. The miRNA expression level is represented in color scale from red (upregulated) to blue (downregulated) in aP-EV compared to PL-EV samples. Three independent pEV batches (*n* = 3), each derived from 50 pooled donors, were analyzed. For each batch or individual sample, two technical replicates were included in the multiomics analyses.

A bioinformatic study of the miRNA microarray results was performed using RStudio software. Of the total number of mature human miRNAs detected, those with a higher differential expression level (*p* < 0.05 and FC > 1.5) were selected (Figure 3A). From this set, a list of top 10 miRNA with superior statistical significance was established for further bioinformatic analysis. Genetic targets were identified for each specific miRNA, with hsa-miR-210-3p, hsa-miR-320a, and hsa-miR-320b featuring the larger number of targets (Figure 3B). The biological significance of individual miRNAs was deduced from the related targets. On one side, GO enrichment analysis revealed four miRNAs with over-represented GO terms according to their associated gene set. Three of these miRNAs (hsa-miR-320a, hsa-miR-320b, and hsa-miR-210-3p) were downregulated in aP-EV compared to PL-EV samples, whereas hsa-miR-4745-5p seemed to be overexpressed in the same context. The hsa-miR-320a was notable for having a total of 547 associated GO terms and for sharing 171 with hsa-miR-320b, while the hsa-miR-210 stand out for having 236 unique GO terms, not found in the remaining miRNAs (Figure 3C). On the other side, KEGG analysis reported biological pathways associated with miRNAs hsa-miR-320b, hsa-miR-320a, and hsa-miR-210-3p, all of them downregulated in aP-EV compared to PL-EV samples (Figure 3D). Moreover, a higher interaction size is also observed between hsa-miR-320b and hsa-miR-320a. These differentially expressed miRNAs were subsequently validated across the entire series using quantitative RT-PCR. The results demonstrated a fold-change of 1.4 between PL-EV and aP-EV, and a fold-change of 14.3 between PL and aP.

**Figure 4:**
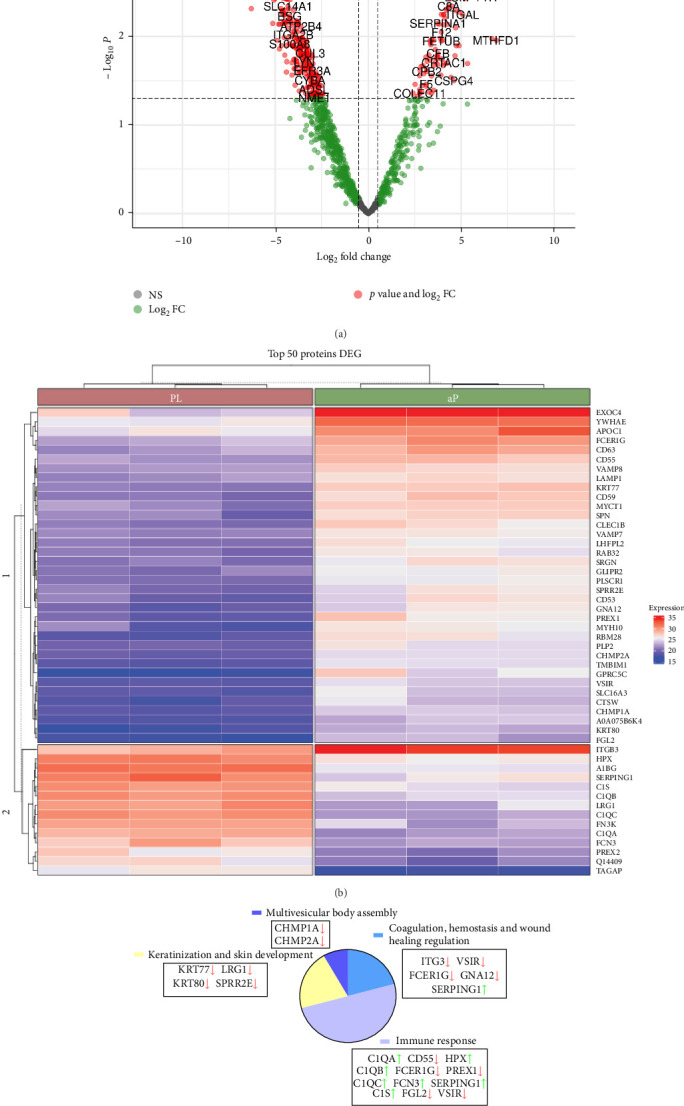
Differential expression analysis of proteins. (A) Volcano plot generated from LC-MS/MS raw data. Each detected protein is plotted according to its statistical significance and fold-change value. Significant up- and downregulated proteins (*p* < 0.05; FC > 1.5) are shown in red dots, comparing PL-EV vs. aP-EV. Proteins with FC < 1.5 are shown as green dots and no significant differences are shown as black dots. (B) Heat map showing *z*-score expression (right scale) of top 50 differentially expressed protein in PL-EV and aP-EV. (C) Diagram of parts showing the biological functions (ORA), enriched in the top 50 most expressed proteins, applied to regenerative medicine and synthesis of extracellular vesicles enriched (green arrow) or downregulated (red arrow) in PL-EV, proteins are sorted by pattern term biological function. Three independent pEV batches (*n* = 3), each derived from 50 pooled donors, were analyzed. For each batch or individual sample, two technical replicates were included in the multiomics analyses.

Differences were also observed between the protein content detected in PL-EV and aP-EV (Figure 4A). These results were filtered to determine the most differentially expressed proteins (*p* < 0.05 and FC > 1.5) and a list of the top 50 proteins in PL-EV and aP-EV was obtained (Figure 4B). From the set of proteins that were in this top 50, a functional enrichment was performed, with 287 and 65 biological functions enriched in over-representation analysis (ORA) and gene set enrichment analysis (GSEA), used to identify whether certain groups of genes or proteins (gene sets) are over-represented in a selected list of genes/proteins and to determine whether a predefined set of genes/proteins shows statistically significant differences between two biological states, respectively. Between these proteins included in the analysis, biological functions were found to be significantly enriched in aP-EV and PL-EV and were also related to their regenerative potential (Figure 4C). Among the genes with enriched ORA biological functions, those whose expression was highest in PL-EV were found to be enriched for the regulation of Wound healing including coagulation and hemostasis processes, as esterase C1 (C1) inhibitor (SERPING1) gene. In addition, the proteins overexpressed in PL-EV, complement C1q A chain (C1QA), complement C1q B chain (C1QB), complement C1q C chain (C1QC), complement serine protease (C1S), and ficolin-1 (FCN1) were highly enriched in processes of immune response regulation. Moreover, those genes overexpressed in aP-EV, which are indicated in Figure 4C were also enriched for the biological functions of wound healing, skin development and multivesicular body assembly and not so enriched for the immune response.

**Figure 5:**
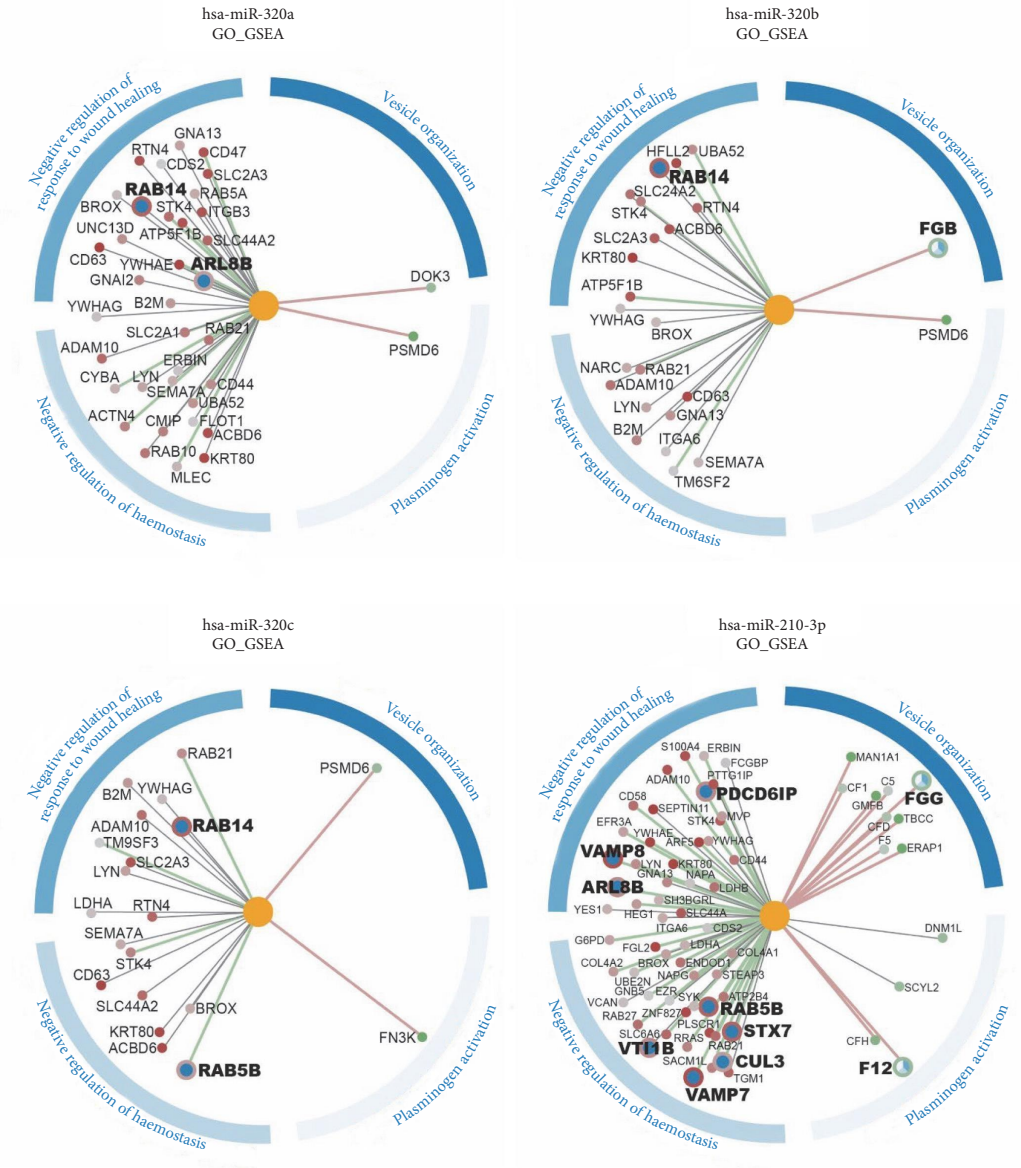
miRNA and proteomic GO GSEA correlation of miRNA overexpressed in PL-EV (miR-320 family and miR 210-3p). The lines connecting genes to miRNAs show the correlation between miRNA and gene expression. The color of the line indicates negative (green), positive (red), or no correlation (gray). The line indicates the degree of correlation. The genes show the value of the fold change in proteomics (red downregulated and green upregulated) in PL-EV vs. aP-EV. The radius of the gene indicates whether it is present in any of the GOs of interest (large radius) or not (small radius). The circle around each gene indicates which GO is involved. Blue outer circle: indicates the selected GO with the color it identifies. Three independent pEV batches (*n* = 3), each derived from 50 pooled donors, were analyzed. For each batch or individual sample, two technical replicates were included in the multiomics analyses.

**Figure 6:**
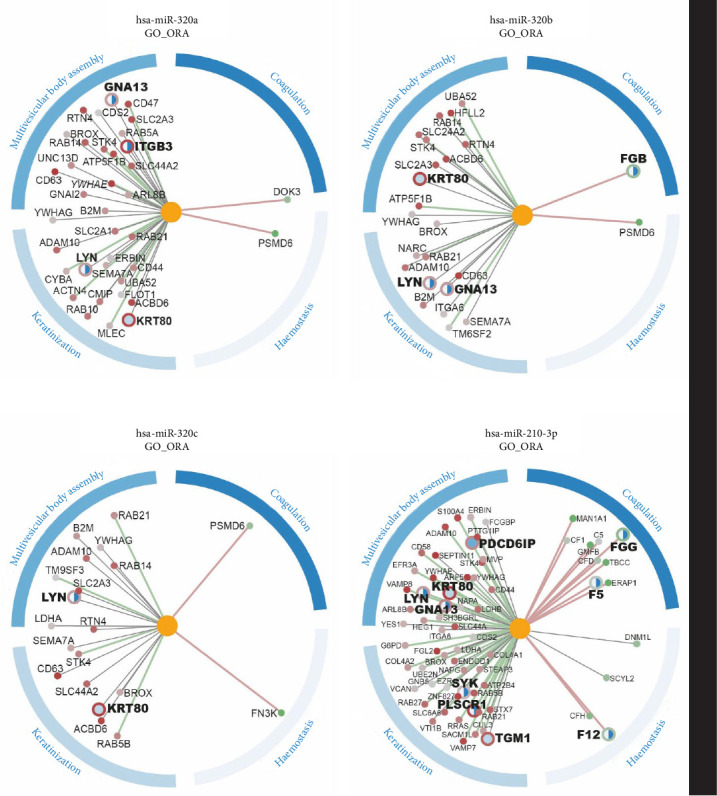
miRNA and proteomic GO ORA correlation of miRNA overexpressed in PL-EV (miR-320 family and miR 210-3p). The lines connecting genes to miRNAs show the correlation between miRNA and gene expression: The color of the line indicates negative (green), positive (red), or no correlation (gray). The dashed line indicates the degree of correlation. The genes show the value of the fold change in proteomics (red down and green up). The radius of the gene indicates whether it is present in any of the GOs of interest (large radius) or not (small radius). The circle around each gene indicates which GO is involved. Blue outer circle indicates the selected GO with the color it identifies. Three independent pEV batches (*n* = 3), each derived from 50 pooled donors, were analyzed. For each batch or individual sample, two technical replicates were included in the multiomics analyses. Significant negative correlations were mostly detected between genes of differentially expressed proteins in proteomics analysis and these PL-EV overexpressed miRNAs, especially in those proteins carried in aP-EV, for instance RAB14, vesicle associated membrane protein 7 (VAMP7), vesicle associated membrane protein 7 (VAMP8), or syntaxin (STX7) in GSEA analysis (Figure 5) or integrin subunit beta 3 (ITGB3), keratin 80 (KRT80), spleen associated tyrosine kinase (SYK), or tyrosine-protein kinase Lyn (LYN) in ORA analysis (Figure 6). Moreover, many of these miR-regulated genes were enriched for GO biological functions, both for GSEA analysis (Figure 5) and ORA analysis (Figure 6).

Interaction analysis between the miRNA of interest with high differential expression in PL-EV and the differentially expressed proteins in the proteomics analysis of PL-EV vs aP-EV for GSEA and ORA enriched biological functions was also evaluated; results show multiple experimental correlations between miRNA overexpressed in PL-EV, expressed as connecting lines with the protein genes and their main functions related to wound healing (Figures 5 and 6).

## 4. Discussion

pEV have demonstrated in preclinical studies to be the most therapeutically potent fraction of platelets secretome [21, 22]. However, there is no consensus neither comparative studies evaluating the optimal source of pEV to be used as an active compound in regenerative medicine. Here, we have compared pEV derived from three different platelet concentrates, confirming that the ones derived from PLs are the best candidate for wound healing treatments. Platelet activation is the basis of EV biological function and for their release. Activation can be triggered by physiological agonists (collagen, thrombin, etc.) [23–26], platelet storage, cryopreservation, or shear stress [27, 28]. Thus, here we evaluated pEV derived from platelet concentrates activated with calcium chloride either just after its obtention (fP-EV) or after 7 days storage (aP-EV); or activated through lysis (PL-EV).

Previous studies have shown that the amount of pEV released by platelets depends on their aggregation state, the length of time they are exposed to room temperature, and the freeze–thaw cycles used in the platelet production process [2]. Apart from the activation method, here we also compare the effect of platelet storage, confirming that a higher yield is obtained the longer the rest time at room temperature, as sustained by previous studies, since the resting time facilitates vesiculation and secretion [29]. In addition, our results confirm that the process of freezing and thawing platelets undergone to obtain PL also implies an increase in the number of particles to be obtained when compared with the activation with CaCl_2_ used for aP and fP. Thus, in this comparative study, we confirm that the characteristics of pEV may vary depending on the method of preparation of the platelet concentrate as pEV source. As already stated, due to the low yield achieved, fP-EV were excluded from further analysis.

The in vitro wound healing assay showed that fibroblast migration was improved when PL-EV were used as treatment. This migration improvement can be crucial in the haemostasis, proliferation, and remodeling phases of the wound healing process. Fibroblast migration is increased in the haemostasis wound healing phase and this cell movement is responsible for the release of mediators by immune cells to the wound area during the inflammatory phase of the wound healing [28, 30]. Additionally, extracellular matrix (ECM) components are orchestrated by fibroblast activity to support reepithelization during the proliferative and remodeling phases of wound healing [31, 32]. Thus, increased fibroblast migration is desirable for treatment of chronic wound healing in which pathological cell environments are related to poor and senescent fibroblast activity [31, 33–35]. Notably, fibroblasts in the treated in vitro conditions displayed altered morphology compared to controls, including increased size, reduced confluence, and greater heterogeneity—features consistent with an activated state linked to tissue repair. This heterogeneity likely reflects transitions toward migratory and contractile phenotypes, supporting the faster wound closure observed in the migration assay [36]. Such morphological changes, including elongation and protrusions, are often accompanied by cytoskeletal remodeling (e.g., actin reorganization and focal adhesion dynamics), enhancing motility [37]. These adaptations are typical of activated or premyofibroblast states involved in reepithelialization and ECM remodeling during wound healing [30].

In this work, we have also performed a multiomics analysis to deeply characterize pEV obtained from PL and from aP. It is worth to remind that pEV molecular content has been postulated, at least in part, as the molecular mechanisms underlying pEV functionality [1, 9, 15]. Here we identified three overexpressed miRNA in PL-EV compared to aP-EV that might be involved in their wound healing activity. Also, a greater number of biological targets were found in the interaction analysis. miR-210-3p and hsa-miR-320 family, highly expressed in PL-EV compared to aP-EV, have shown to regulate different targets involved in wound closure. For instance, the miR hsa-miR-210-3p has been shown to enhance keratinocytes activity and to promote artery remodeling in uterine spiral artery [38, 39]. Moreover, the miR-320 family, in which hsa-miR-320a y hsa-miR-320b are included, are related to re-epithelization and intestinal mucosa wound healing, having been identified genes ATP-binding cassette subfamily A (ABCA2), actin beta-like 2 (ACTBL2), or aminoacyl tRNA synthetase complex interacting multifunctional protein 1 (AIMP1) as their molecular targets [40]. This function related to wound healing agrees with the increased cell migration results observed in the wound healing assay for PL-EV. It should also be noted that hsa-miR-320a has been described as one of the most highly expressed miRNAs in platelet concentrates and its expression related to the one of has-mir-127 has been suggested as a marker to assess the “validity period” of platelet concentrates [41].

The protein analysis result validates that the preparation method, the state of activation and storage of the original platelet concentrate determines the protein cargo of pEVs [27, 42]. However, it is noteworthy that out of the set of 287 and 65 biological functions enriched in ORA and GSEA, respectively, a higher number of overexpressed proteins related to enriched functions involved in wound healing processes were found in aP-EV. This observation may present a contrast with the findings from in vitro experiments and miRNA differential expression analysis. The elevated expression of some proteins such as charged multivesicular body protein 1A (CHMP1A) and charged multivesicular body protein 2A (CHMP2A) in aP-EV, comparted with PL-EV, whose enriched function is to intervene in the assembly of multivesicular bodies may suggest that aP-EV may be more immature vesicles or released endosomes and that they have not been able to carry out a correct interaction with cells, such as PL-EV [43, 44]. PL-EVs may display a cargo profile and surface composition that is more favorable for functional delivery and interaction with recipient cells, compared to aP-EVs. These differences could reflect variations in vesicle biogenesis, platelet activation status, or processing methods, resulting in enhanced bioactivity despite similar or even lower total protein abundance.

The multiple experimental correlations between miRNA overexpressed in PL-EV and the protein content have previously been experimentally related to their regenerative potential, for the plasminogen activation function, negative regulation of hemostasis, negative regulation to wound and vesicle organization for GSEA, and keratinization, multivesicular assembly, coagulation, and regulation of wound healing process [45–47].

The collected data supports PL as the most suitable candidate for clinical translation. Notably, a higher EV yield is obtained from PL compared to aP, which enhances scalability and standardization for therapeutic applications. In contrast, the 1-week resting period required for aP preparation results in a marked reduction in EV yield and particle concentration, likely due to vesicle degradation during storage. Beyond quantity, PL-EVs also demonstrate superior functional activity, with a molecular cargo—particularly the microRNA profile—more effectively promoting wound healing responses in vitro. These combined advantages highlight the translational potential of PL-EVs over aP-EVs in regenerative medicine.

Taking this into account, it is possible to have more clarity on the potential in regenerative medicine of pEV depending on their source (PL or aP), with the in silico evaluation of the interaction that may exist between the differential miRNA and protein content as a whole in both PL-EV and aP-EV groups. The previously described important role of miRNAs (miR-210-3p and has-miR-320 family) in the gene regulation of the wound healing process is well known [38–40]. In the same way, pEV cargo proteins identified in the proteomics analysis are mostly regulated by these miRNAs. Our results show improved wound healing in PL-EV treated cells, which can be explained to a greater extend by their miRNA content rather than their protein content. This provides some insight into the underlying molecular mechanisms of the cargo delivered to cells when aP-EV and PL-EV are used as a treatment, and thus, their potential in regenerative medicine. Overall, in line with previous studies suggesting that miRNAs can achieve a much more profound, long-lasting, and stable regulation of gene expression compared to proteins [48], in this line, the effect of EV is mostly attributed to miRNAs [1]. Likewise, our results show that the method of preparation of the platelet concentrate determines the molecular cargo of the obtained pEV, and, in turn, their functionality as we show in the in vitro functional studies.

## 5. Conclusion

In conclusion, our results demonstrate that PL is the most suitable platelet concentrate candidate for pEV isolation to reach the clinic for acute and chronic wound healing. We have identified hsa-miR-320a, hsa-miR-320b, and hsa-miR-210-3p as potential molecules underlying the molecular mechanisms responsible for mediating wound healing. Our results also show that the method of preparation of the platelet concentrate can also determine the molecular cargo of pEVs, and, in turn, their functionality, as we show in the in vitro studies.

## Data Availability

We have submitted all experimentally relevant data of our experiments to the figshare repository (https://doi.org/10.6084/m9.figshare.26499286).

## Supporting Information

Supporting Information SEC characterization and whole western blot membranes are fully displayed in a side document as supporting information.
