# Supplementary material for: A Multiomic Study of Platelet-Derived Extracellular Vesicles and Impact of Platelet Concentrate Sources
Source: IET Nanobiotechnol. 2025 Aug 19;2025:8358424. doi: 10.1049/nbt2/8358424 (PMC12380512; doi:10.1049/nbt2/8358424)
Supplement: Supporting Information — SEC characterization and whole western blot membranes are fully displayed in a side document as supporting information. [file 8358424.f1.docx]

***Supplementary Material***

***A Multiomic Study of Platelet-derived Extracellular Vesicles and Impact of Platelet Concentrate Sources.***

*Andreu Miquel Amengual-Tugores^1,2,3^ ‡; Carmen Ráez-Meseguer^1,2,3^ ‡; Maria Antònia Forteza-Genestra^1,2,3^; Javier Calvo^1,2,4^; Antoni Gayà ^1,2,4^; Marta Monjo^1,2,3*^; Joana Maria Ramis^1,2,3*^.*

1. [Group of Cell Therapy and Tissue Engineering (TERCIT](https://tercit.uib.es/)[)](https://tercit.wordpress.com/), Research Institute on Health Sciences (IUNICS), University of the Balearic Islands, Palma, Spain.
2. [Health Research Institute of the Balearic Islands (IdISBa),](http://www.idisba.es/cat/) Palma, Spain.
3. [Department of Fundamental Biology and Health Sciences,](https://www.uib.cat/lauib/Govern-i-organitzacio/estructura/Departaments/dbf/) University of the Balearic Islands, Palma, Spain.
4. Fundació Banc de Sang i Teixits de les Illes Balears (FBSTIB), Palma 07004, Spain.

‡ These authors contributed equally to this work.

* Correspondence: [marta.monjo@uib.es](mailto:marta.monjo@uib.es) ;Tel.: +34 971 25 99 60; [joana.ramis@uib.es](mailto:joana.ramis@uib.es); Tel.: +34 971 17 23 76.

**
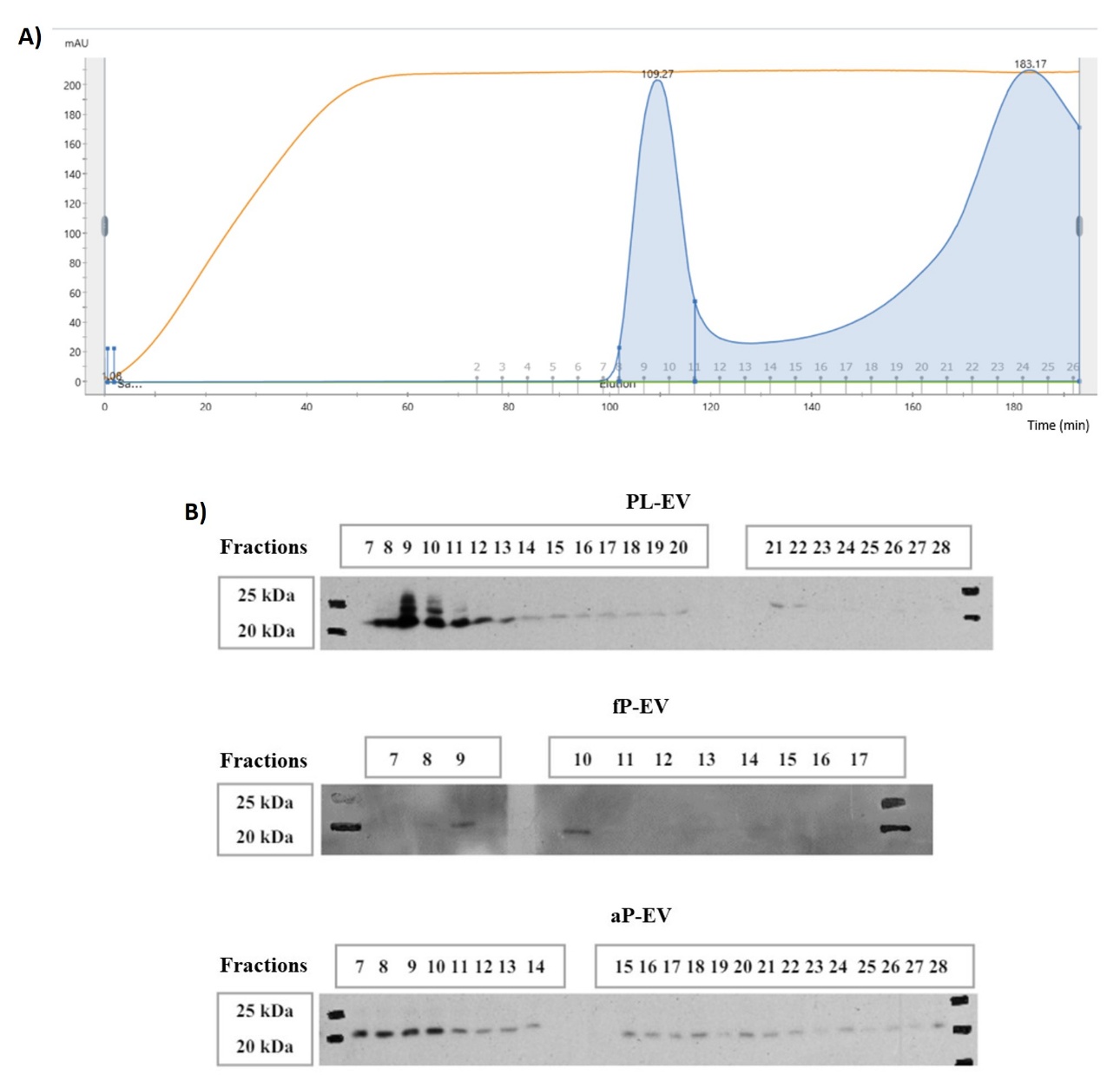
**

**Supplementary figure 1**. SEC fractions characterization. A) SEC chromatogram at an absorbance of 280 nm (blue line and area), the orange line indicates conductivity of the solute, and the X axis indicates the elution time. Fractions from 1 to 26, 5 mL each, were collected for characterization. B) Western Blot of SEC fractions; CD9 enrichment in fractions 8,9,10, which were used for the pEV pools from PL-EV, fP-EV and aP-EV. CD9 were detected using anti-human CD9 monoclonal antibody (clone Ts9 diluted 1:2,000, Thermo Fisher) and HRP-coupled secondary antibody (Thermo Fisher) diluted 1:2,000.


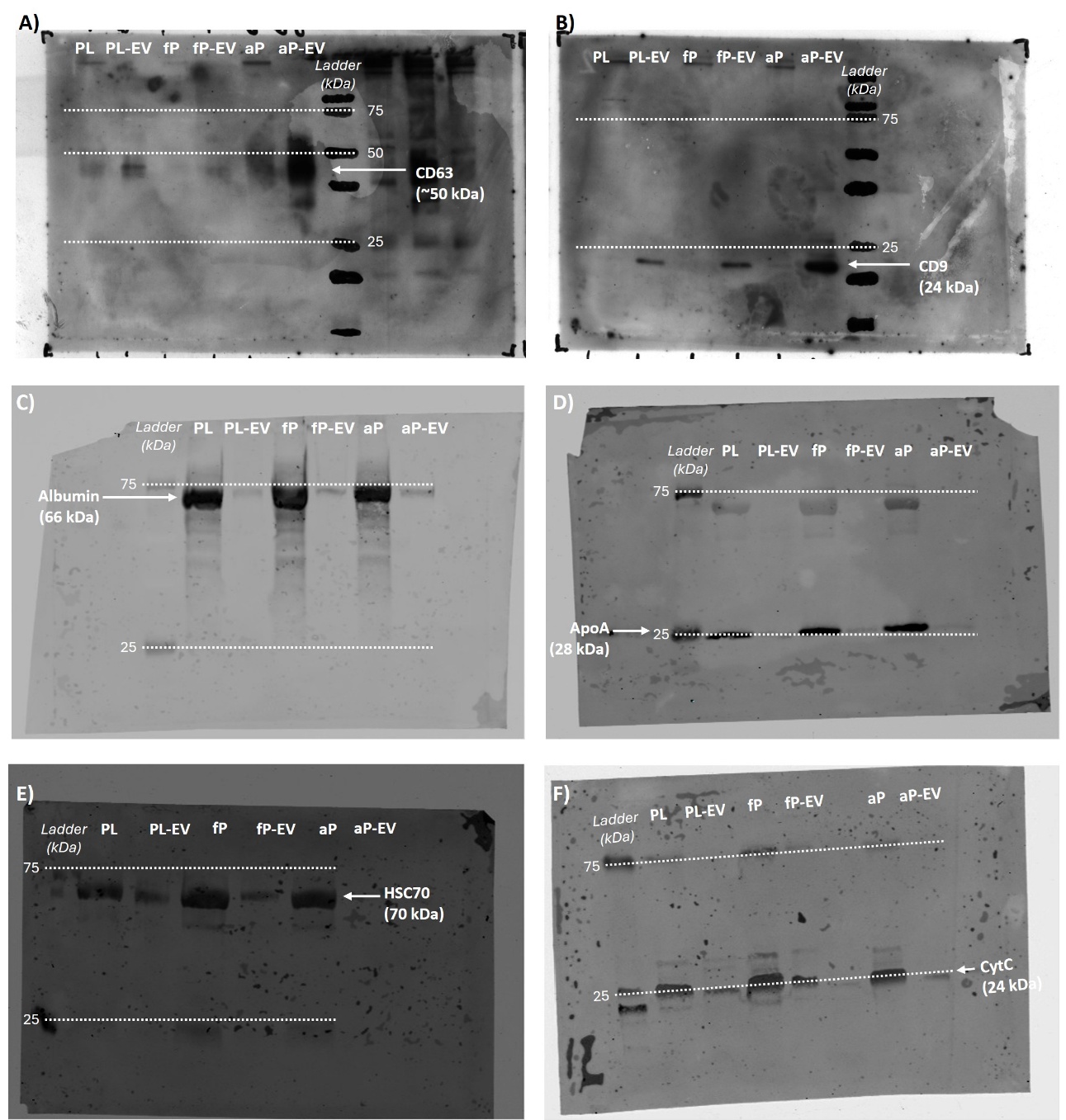


**Supplementary figure 2**. Western blot of whole membranes for pEV characterization according to MISEV2023 guidelines. Detection of the following markers: A) CD63, B) CD9, C) Albumin, D) ApoA, E) HSC70, and F) Cytochrome C (CytC), as recommended by MISEV2023. pEV samples and their corresponding sources are indicated.
